# Exercise intensity influences variability and test-retest reliability of pulmonary gas-exchange measurements during constant work-rate cycling

**DOI:** 10.3389/fspor.2026.1814527

**Published:** 2026-06-22

**Authors:** Alessandro L. Colosio, Massimo Teso, Maura Loi, Lena Stuer, Jan Boone, Silvia Pogliaghi

**Affiliations:** 1 Laboratoire Interuniversitaire de Biologie de la Motricité, Université Jean Monnet Saint-Etienne, Saint-Étienne, France; 2 College of Health and Life Sciences, Hamad Bin Khalifa University, Doha, Qatar; 3 Department of Neurosciences, Biomedicine and Movement Sciences, University of Verona, Verona, Italy; 4 Department of Movement and Sports Sciences, Ghent University, Ghent, Belgium; 5 Department of Clinical and Experimental Medicine, University of Foggia, Foggia, Italy

**Keywords:** carbon dioxide, CORTEX metaLyzer 3B, COSMED quark B2, indirect calorimetry, oxygen consumption, V̇o2

## Abstract

**Introduction:**

The study examined how exercise intensity and participants' characteristics influence the variability and test-retest reliability of pulmonary gas-exchange while providing domain-specific reliability benchmarks for exercise testing.

**Methods:**

Forty-two healthy adults (V̇O_2max_ = 49.6 ± 8.8 ml·kg_-1_·min_-1_) completed a ramp incremental exercise test and six 6-min constant work-rate cycling trials distributed across moderate, heavy, and severe exercise intensities. Exercise intensities were set at 33%Δ and 66%Δ between baseline and gas exchange threshold (GET), GET and respiratory compensation point (RCP), and RCP and V̇O2max, respectively. Pulmonary oxygen uptake (V̇O_2_), carbon dioxide output (V̇CO_2_), and ventilation (V̇E) were recorded using two metabolic carts. Systematic differences between days and intensities were assessed with two-way repeated-measures ANOVA. Variability and participants' characteristics were examined with within-trial coefficient of variation (CV%), Pearson correlations, linear mixed-effects models (LMME), intraclass correlation coefficient (ICC), standard error of measurement (SEM), minimal detectable change (MDC), and Bland-Altman analysis.

**Results:**

V̇O_2_, V̇CO_2_, and V̇E increased with intensity (*p* ≤ 0.001), with no day-to-day differences (*p* > 0.05). CV% decreased progressively with intensity, from ∼7-10% in the moderate to <5% for V̇O_2_ and V̇CO_2_ in the severe domain. ICCs were excellent (>0.95) in the heavy and severe domains. Correlations showed moderate negative associations between CV% and aerobic fitness (*r* -0.34 and -0.58, *p* < 0.05), while LMME identified exercise intensity as the main factor associated with variability. MDC for V̇O_2_ ranged from 166 to 415 ml·min_-1_, and Bland-Altman plots confirmed minimal bias.

**Discussion:**

exercise intensity influences the variability and reliability of breath-by-breath gas-exchange responses, with relative variability decreasing at higher metabolic loads. Fitness-related differences in CV% possibly reflect physiological scaling effects. These results provide practical reliability thresholds and improve the interpretation of repeated-measures testing in exercise physiology.

## Introduction

Breath-by-breath gas-exchange measurements provide a non-invasive means to assess whole-body oxygen uptake (V̇O_2_) during exercise. V̇O_2_ reflects the integrated response of the respiratory, cardiovascular, and muscular systems, and therefore represents a global measure of oxygen transport and utilisation (1–3) that gained significant interest in experimental and applied physiology. The increasing accessibility of metabolic carts has supported the widespread use of breath-by-breath gas-exchange assessment across clinical and performance settings (4, 5). In clinical practice, cardiopulmonary exercise test-derived variables are used to evaluate functional capacity, inform perioperative risk stratification, and guide exercise prescription in rehabilitation (6, 7). In sport performance, they provide a basis for tracking aerobic capacity, detecting training-induced adaptations, and refining the structure of conditioning programs across intensity domains (4, 5).

Despite the central role of breath-by-breath gas exchange in exercise testing, the variability and reliability of these measurements are influenced by multiple interacting factors. From an applied perspective, accurate and reproducible estimates of pulmonary gas exchange are essential for clinical decision-making, evaluation of interventions, and classification of individuals in both health and performance settings (6). Physiological variability arises from factors such as breathing pattern, day-to-day biological fluctuations, and lifestyle influences (e.g., diet or prior activity), and interacts with technical and procedural sources of error. These include analyser precision, calibration procedures, signal and data processing choices (8). Moreover, pulmonary gas exchange variables such as V̇O_2_ and V̇CO_2_ are not directly measured, but derived from primary measurements of expired gas fractions (FEO_2_, FECO_2_) and ventilation (9), together with assumptions related to the breathing cycle (10). As such, variability in the measured signals propagates into the calculated variables, adding an additional layer of uncertainty. Trying to quantify systems accuracy, researchers have investigated metabolic carts using different approaches including comparisons between devices and the Douglas bags (11–16). More recently, studies using both human and metabolic simulators have demonstrated that variability can result in between-day or between-device errors for V̇O_2_ and V̇CO_2_ exceeding 5% (17–19). Despite this, these derived variables remain the primary outcomes used in both clinical and applied physiology, reinforcing the need to better characterise their variability and reliability under different exercise conditions.

Among the factors affecting variability and test–retest reliability, exercise intensity and individual characteristics (i.e., fitness level) remain incompletely described. As workloads increase, metabolic and ventilatory responses become larger and more stable, potentially improving the signal-to-noise ratio of breath-by-breath measurements. Conversely, at lower intensities (e.g., moderate domain of exercise), smaller response amplitudes and greater relative biological noise may increase variability. Previous research has shown that ventilatory and metabolic variability changes across intensity domains, with slower adjustment kinetics and a progressive loss of efficiency in the heavy domain due to the V̇O_2_ slow component, and a failure to reach steady state in the severe domain (20–22), further complicating the correct interpretation of metabolic carts variables. Understanding how exercise intensity and participants’ characteristics influence the variability of breath-by-breath gas-exchange responses is therefore essential for accurate interpretation of exercise testing data, informing study design, and improving confidence in clinical and research outcomes (23–25).

Accordingly, this study examined the day-to-day variability and test-retest reliability of pulmonary V̇O_2_, V̇CO_2_, and ventilation (V̇E) across the full exercise intensity continuum (i.e., moderate, heavy, and severe exercise domains). We hypothesised that higher intensities would yield smaller physiological variability and thus higher reliability. In addition, we explored whether individual characteristics could influence the variability of these measurements. A secondary objective was to provide domain-specific reliability estimates that can guide the design and interpretation of repeated-measures studies in exercise physiology.

## Methods

### Ethical approval

All procedures performed in this study were in accordance with the 1964 Helsinki Declaration and its later amendments or comparable ethical standards, apart from registration in a database. The subjects were fully informed of any risk and discomfort associated with the experiments before giving their written consent to participate in the study. The protocol was reviewed and approved by the University of Verona Ethics Committee for Research on Human Subjects (CARU no. 15/2019) and complied with the Declaration of Helsinki.

### Participants

Forty-two active participants (20 women, age 26.0 ± 4.3 years, body mass 68.4 ± 10.2 kg, height 173.5 ± 9.0 cm, maximal oxygen uptake (V̇O_2max_) 49.6 ± 8.8 ml·kg^−1^·min^−1^) were included in this study. Inclusion criteria were an age between 20 and 35 years and performing recreational aerobic activity (e.g., cycling or running) at least 3–4 times per week, with sessions lasting ≥30 min. Participants were recreationally active but not involved in competitive endurance sports. Exclusion criteria were smoking and any medical condition or therapy that could influence physiological responses during testing or compromise participant safety (e.g., cardiovascular disease, asthma).

### Protocol

A within-subject crossover design was adopted, each participant completed a medical screening followed by anthropometric measurements (Body mass using a digital scale, Seca877, Seca, Leicester, UK, and height using a vertical stadiometer, Seca, Leicester, UK) and a ramp incremental test to exhaustion to determine the gas exchange threshold (GET), respiratory compensation point (RCP) and V̇O_2max_. Then, they completed twelve constant work rate (CWR) tests on six separate days, with two CWR tests per day. Each CWR started with 1 min of seated rest on the ergometer, followed by 3 min unloaded cycling at 30 revolutions per minute (rpm) performed to standardise the exercise onset. Thereafter, work rate was immediately increased to the target power output and maintained for 6 min. Six different intensities were chosen, two were in the moderate intensity domain (M1 and M2), two in the heavy intensity domain (H1 and H2) and two in the severe intensity domain (S1 and S2), as follows:

- Moderate intensity trials: 33% (M1) and 66% (M2) of the difference between V̇O_2_ at rest and GET
- Heavy intensity trials: 33% (H1) and 66% (H2) of the difference between V̇O_2_ at GET and the RCP
- Severe intensity trials: 33% (S1) and 66% (S2) of the difference between V̇O_2_ at RCP and V̇O_2max_

And combined as:

- M1—20 min of seated rest—S2
- M2—20 min of seated rest—S1
- H1—20 min of seated rest—H2

This structure was chosen to contain the total number of testing sessions, while at the same time minimising potential carry-over effects between CWR trials. Combinations were executed in randomised order and were performed twice to assess test–retest reliability. All procedures were completed within a maximum of 6 weeks and with at least 2 full days between sessions. Participants were instructed to maintain their habitual physical activity patterns throughout the study period and were informally monitored regarding changes in training routine besides the testing sessions. Although formal longitudinal monitoring of training was not performed, the randomised order of sessions and the within-subject design reduce the likelihood that minor fluctuations in fitness systematically influenced the reliability outcomes. During all testing sessions, breath-by-breath gas-exchange and ventilation were continuously recorded in breath-by-breath mode with two regularly maintained metabolic carts: twenty-two participants (10 women) using a CORTEX MetaLyzer 3B (CORTEX Biophysik, Leipzig, Germany) and the other twenty (10 women) using a COSMED Quark b2 (COSMED, Rome, Italy). In the COSMED system, O₂ and CO₂ concentrations were measured using paramagnetic and nondispersive infrared analysers, respectively. In the MetaLyzer 3B, O₂ concentration was measured using a chemical fuel-cell sensor and CO₂ concentration using a nondispersive infrared analyser. Ventilation was quantified through turbine-based flow sensors in both systems. Both systems were calibrated before each test according to manufacturer recommendations using certified calibration gases and flow calibration procedures. Heart rate (HR) was monitored continuously using a chest strap sensor (H7 Sensor, Polar, Kempele, Finland). All trials were conducted on an electromagnetically braked cycle ergometer (Sport Excalibur, Lode, Groningen, Netherlands), at a similar time of the day in an environmentally controlled laboratory (18°C, 55%–65% relative humidity). All participants were asked to avoid caffeine intake in the 8 h prior to the tests and to abstain from physical activity at least 24 h before each trial. In order to reduce variability of glycogen and glucose oxidation participants consumed a standardised meal two hours before all the testing sessions (i.e., 2 kg^−1^ of low glycaemic index carbohydrates, 0.5 L of water in the 90 min before testing) (26).

### Ramp incremental test

Before the test, the position of the saddle and the handlebar of the ergometer was adjusted individually and noted to be used during all subsequent tests. The ramp incremental test consisted of a 4 min baseline cycling at 50 watts, followed by a 15–30 watt·min^−1^ increase in power output until volitional exhaustion. The ramp incremental protocol was preceded by a 6 min CWR at 80 (women) or 100 (men) watts to calculate the mean response time for each subject (27).

During the ramp, participants were asked to choose a self-selected cadence between 70 and 90 rpm and to maintain it during the entire session and the successive visits. The test was terminated when the subject was incapable of sustaining the selected cadence in a range of 5 rpm for more than 5 s despite strong verbal encouragement.

Maximal effort was confirmed using established criteria, including the attainment of a V̇O_2_ plateau despite increasing work rate, a respiratory exchange ratio ≥1.10, heart rate within 10 beats·min^−1^ of age-predicted maximum, and volitional exhaustion. Gas analysers were calibrated prior to each test using certified calibration gases according to manufacturer recommendations, and flow sensors were calibrated using a 3 L syringe. All tests were supervised by experienced exercise physiologists trained in cardiopulmonary exercise testing procedures. The obtained breath-by-breath data were treated by removing data points deviating 3 standard deviations (SD) from the local mean. Then, they were linearly interpolated on a 1 s basis and averaged every 5 s. V̇O_2max_ and V̇E_peak_ was determined as the highest V̇O_2_ obtained over a 10 s interval (28). GET and RCP were identified with the standard technique from gas exchange variables by three blinded expert reviewers. Reviewers were trained exercise physiologists with formal academic training in exercise science. Three reviewers were at postdoctoral or professorial level with multiple peer-reviewed publications using gas-exchange threshold methodology, and two were PhD candidates with ≥1.5 years of experience in the exercise physiology laboratory and at least three publications in related areas. Briefly, GET was determined by visual inspection using an integrated approach including the V-slope method, ventilatory equivalents, and end-tidal gas pressures. Specifically, GET was identified as the point at which V̇CO_2_ began to increase disproportionately relative to V̇O_2_, accompanied by a systematic increase in V̇E/V̇O_2_ and end-tidal PO_2_ (PETO_2_) without a concomitant increase in V̇E/V̇CO_2_ or decrease in end-tidal PCO_2_ (PETCO_2_) (29). RCP was identified as the point at which PETCO_2_ began to decrease following a period of isocapnic buffering, together with a systematic increase in V̇E/V̇CO_2_ and a second disproportionate increase in the V̇E-to-V̇O_2_ relationship (30). This point was confirmed by examining V̇E/V̇CO_2_ plotted against V̇O_2_ and by identifying the second breakpoint in the V̇E-to-V̇O_2_ relation. Thresholds identifications were first performed independently and blinded, then averaged. In cases where one reviewer differed by ≥150 ml·min^−^¹ in V̇O_2_ from the other two, the threshold was re-examined and consensus was reached through joint discussion. These values were used to determine the intensities of successive tests.

### Constant work rate tests

After the first ramp test sessions, participants performed twelve CWR with the same bike fitting and cadence used for the ramp incremental test. The CWR to the specific V̇O_2_ targets in the moderate, heavy, and severe domains was determined at 33%*Δ* and 66%*Δ* between baseline and GET, between GET and RCP and between RCP and V̇O_2max_, respectively. For this purpose, the V̇O_2_/W relationship identified with the incremental test was shifted to the left to account for the individual mean response time and slow component (31). Below GET, the correction consisted solely of shifting the ramp-derived V̇O_2_/W relationship to the left according to the individual MRT, accounting for the delayed adjustment of pulmonary V̇O_2_ during incremental exercise. Although MRT has been reported to vary across exercise intensities rather than representing a single physiological constant (32), this approach was used pragmatically to improve the estimation of constant-work-rate exercise intensity from ramp incremental exercise compared with direct extrapolation alone. For exercise intensities above GET, an additional correction was applied to account for the altered V̇O_2_/W relationship associated with the development of the V̇O_2_ slow component during constant-work-rate exercise. This approach is based on the observation that the V̇O_2_/PO relationship remains approximately linear but with a steeper slope above GET during constant-load exercise, whereas this change is only partially expressed during ramp exercise (33).

For each CWR exercise, respiratory data were treated with the same methodology as for the ramp incremental test (interpolation, 5 s averages), and analysed over the final minute of each stage. This approach was preferred over raw breath-by-breath data to standardise sampling density across exercise intensities, where breathing frequency differs substantially to enable consistent comparison of variability metrics. Within this time window, both within-trial variability and test-retest metrics were quantified (see statistics). Importantly, variability metrics were derived from all 5 s data points within this time window and not from time-averaged values.

### Statistics

Independent sample *T*-tests were used to examine possible differences in anthropometrics and functional characteristics in the two groups of participants who performed the tests with CORTEX or COSMED metabolic carts.

After verifying the assumptions for repeated-measures analysis, two-way repeated-measures ANOVAs were conducted separately for each group of participants to assess the effect of intensity and day on V̇O_2_, V̇CO_2_ and V̇E across different cycling intensities using the 1 min averaged values obtained within the final minute of each CWR trial. Before analysis, the normality of the residuals (i.e., the within-subject differences between days) was assessed for each intensity and variable using the Shapiro–Wilk test. Where the assumption of sphericity was violated, Greenhouse–Geisser corrections were applied.

Two complementary levels of analysis were considered: within-trial variability derived from the processed signal, and between-day reliability assessed from time-averaged values.

The coefficient of variation (CV%) was calculated for each participant and condition based on the interpolated and 5 s averaged signal within the final minute (i.e., minute 6) of each constant work-rate trial on both Day 1 and Day 2. For each variable (V̇O_2_, V̇CO_2_, V̇E), CV% was computed from the standard deviation and the mean of all data points within this time window. The resulting CV% represents within-trial physiological and technical variability of the processed breath-by-breath–derived signal during intensity domains approaching a quasi-steady condition (moderate and heavy) or during end-exercise in domains where a steady state is not expected (severe), thereby incorporating any ongoing physiological drift. SD and CV% derived as described above were used as indices of absolute and relative within-trial variability, respectively.

First, Pearson correlations were computed to examine possible associations between participants' characteristics (BMI, absolute and body-mass-normalised V̇O_2max_, and V̇E_peak_) and indices of absolute (SD) and relative (CV%) variability in V̇O_2_, V̇CO_2_, and V̇E during the last minute of exercise at different intensity domains. For this purpose, the four measurements per domain (two intensities × two days) were averaged to yield a single domain-specific value per participant for each variability metric (SD and CV%). Second, linear mixed-effects models were used to quantify the independent effects of exercise intensity and participant characteristics on absolute (SD) and relative (CV%) variability. Exercise intensity was expressed as the percentage of each participant's respiratory compensation point (%RCP) and modelled as a continuous variable to analyse variability across the full intensity spectrum without imposing categorical boundaries. Fixed effects included %RCP, normalised V̇O_2max_, and sex (categorical). Participant was entered as a random intercept to account for repeated observations across days and intensities. BMI, absolute V̇O_2max_, and V̇E_peak_ were excluded *a priori* to minimise collinearity. Models were estimated using restricted maximum likelihood. Model assumptions were evaluated by inspection of residual distribution, homoscedasticity, time-order dependence, and convergence diagnostics. This dual approach allowed us to distinguish cross-sectional associations between individual traits and variability (correlations) from within-subject effects of intensity.

All test–retest reliability metrics were calculated from the 1 min averaged values obtained within the final minute of each stage. Test–retest reliability for V̇O_2_, V̇CO_2_, and V̇E variables was evaluated using the intraclass correlation coefficient (ICC). ICC was interpreted as follows: poor (<0.5); moderate (0.5–0.75); good (0.75–0.9); excellent (>0.90) (34). Moreover, the standard error of measurement (SEM) and minimal detectable change (MDC) were calculated to represent, respectively, the absolute typical error and the threshold beyond which a change in respiratory variables can be interpreted as real. Bland–Altman plots were used to further evaluate the agreement between these measurements (35). The mean bias, SD and 95% limits of agreement (LoA) were calculated, and a one-sided z-test on the bias was performed to test if it was significantly different from zero.

Sample size justification was based on expected variability in steady-state gas-exchange measurements. Previous work has reported breath-by-breath variability corresponding to −2%–3% of V̇O_2_ during steady-state exercise (36), with minimal detectable changes in the range of −100–170 ml·min^−1^ for V̇O_2_ values between −2.0 and 3.5 L·min^−1^. Based on these estimates, a minimum sample size of approximately 6 participants was considered sufficient to achieve 80% power for detecting changes of this magnitude under repeated-measures conditions. The present sample sizes (*n* = 20–22 per group) therefore provide adequate power for the analyses performed. In addition, the repeated-measures design across multiple intensities and days further increases the statistical sensitivity of the study.

Data are presented as means ± SD. All statistical analyses were performed using OriginPro 2025 (OriginLab Corporation, USA) and *α* was set at the 0.05 level. Statistical significance was accepted when *p* < *α*.

## Results

**Figure 1:**
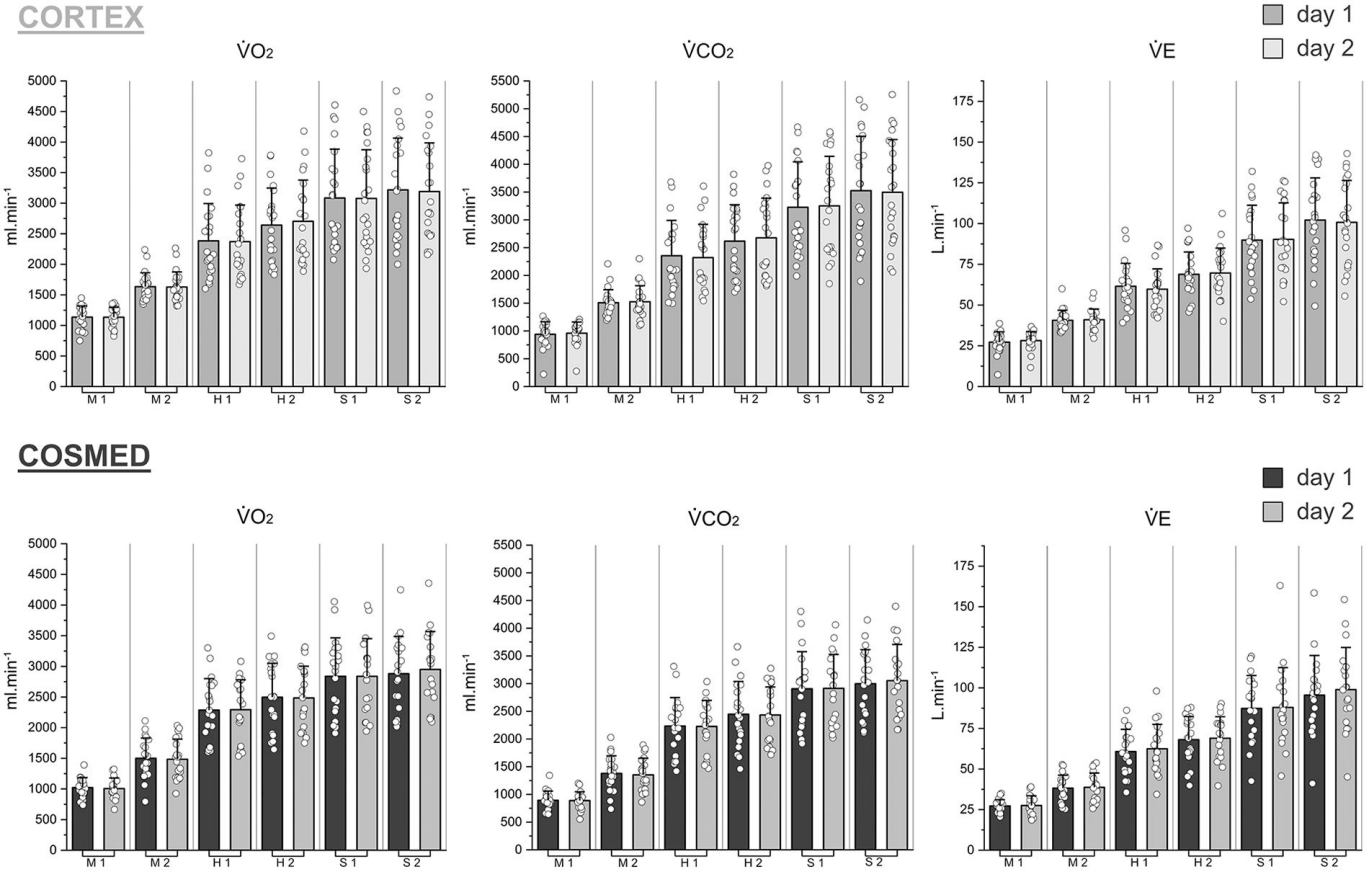
Histograms represent the mean ± SD values recorded during the last min of cycling in the groups tested with the CORTEX metaLyzer 3B (top) and the COSMED quark B2 (bottom) at different intensities on two different days. Dots represent individual values. Two-way repeated-measures ANOVAs revealed a main effect of intensity for all variables (*p* < 0.001), with no consistent effect (except for V̇E COSMED *p* = 0.04) of day and no intensity × day interaction (see Supplementary Table S1 for full results).

Participants' anthropometric and functional characteristics are reported in Table 1. Mean ± SD and individual respiratory responses are displayed in Figure 1. All participants completed all trials, except one participant in the COSMED subgroup who did not complete S2; this observation was excluded from that specific condition only. ANOVAs revealed a main effect of intensity for all respiratory variables (all *p* ≤ 0.001), with no consistent effect of day and no intensity × day interaction. A possible effect of day was observed for V̇E in the COSMED group (*p* = 0.04), but no systematic pattern was evident across variables or systems. Overall, these results indicate increasing physiological responses with intensity and no systematic day-to-day difference. Full output is presented in the Supplementary Table S1.

**Table 1:** Overview of anagraphic, anthropometric, and functional characteristics values are expressed as mean ± SD. Age, height, weight, body mass index (BMI), maximal oxygen consumption (V̇O_2max_), gas exchange threshold (GET), and respiratory compensation point (RCP).

Group	#	Age	Height	Weight	BMI	V̇O_2max_	GET	RCP
		(yrs)	(cm)	(kg)	(kg ⋅ m^-2^)	(ml ⋅ min^-1^⋅Kg^-1^)	%V̇O_2max_	%V̇O_2max_
CORTEX	22 (10♀)	26.3 ± 5.2	174.9 ± 8.2	68.6 ± 7.4	22.4 ± 1.5	51.5 ± 9.8	62 ± 6	84 ± 6^a^
COSMED	20 (10♀)	25.6 ± 3.0	172.0 ± 9.8	68.1 ± 12.9	22.9 ± 2.6	47.5 ± 7.3	65 ± 5	87 ± 3^a^
Mean ± SD	42 (20♀)	26.0 ± 4.3	173.5 ± 9.0	68.4 ± 10.2	22.6 ± 2.1	49.6 ± 8.8	63 ± 5	86 ± 5

a Indicates statistical difference from the other group.

Within-trial CV% values (Table 2) decreased progressively with increasing intensity for all variables. The highest relative variability was observed in the moderate domain (e.g., up to 11.7% for V̇E), whereas at higher intensities CV% values were generally <5% for V̇O_2_ and V̇CO_2_ and <7% for V̇E. This pattern indicates a progressive reduction in relative signal dispersion as metabolic rate increased, and reflects the variability of the processed signal within each stage.

**Table 2:** Mean within-trial coefficient of variation (CV%) during the final minute of exercise across intensities.

		Group CORTEX			Group COSMED		
		V̇O_2_	V̇CO_2_	V̇E	V̇O_2_	V̇CO_2_	V̇E
M1	Day1	8.1%	7.6%	9.1%	5.6%	5.5%	6.1%
	Day2	7.5%	7.9%	8.8%	7.1%	7.1%	7.2%
M2	Day1	7.1%	7.1%	7.9%	4.9%	4.8%	5.3%
	Day2	10.3%	9.4%	11.4%	5.2%	5.0%	6.3%
H1	Day1	4.0%	4.3%	5.7%	3.5%	3.3%	4.4%
	Day2	4.3%	4.6%	6.5%	4.2%	4.1%	5.6%
H2	Day1	3.9%	4.2%	5.8%	3.4%	3.1%	4.0%
	Day2	4.3%	5.0%	7.6%	3.5%	3.2%	4.4%
S1	Day1	3.4%	3.7%	5.9%	2.6%	2.7%	3.7%
	Day2	3.3%	3.5%	5.1%	3.1%	3.0%	4.2%
S2	Day1	3.4%	3.7%	5.9%	2.5%	2.3%	4.1%
	Day2	3.2%	3.3%	5.1%	2.8%	2.7%	4.6%

**Figure 2:**
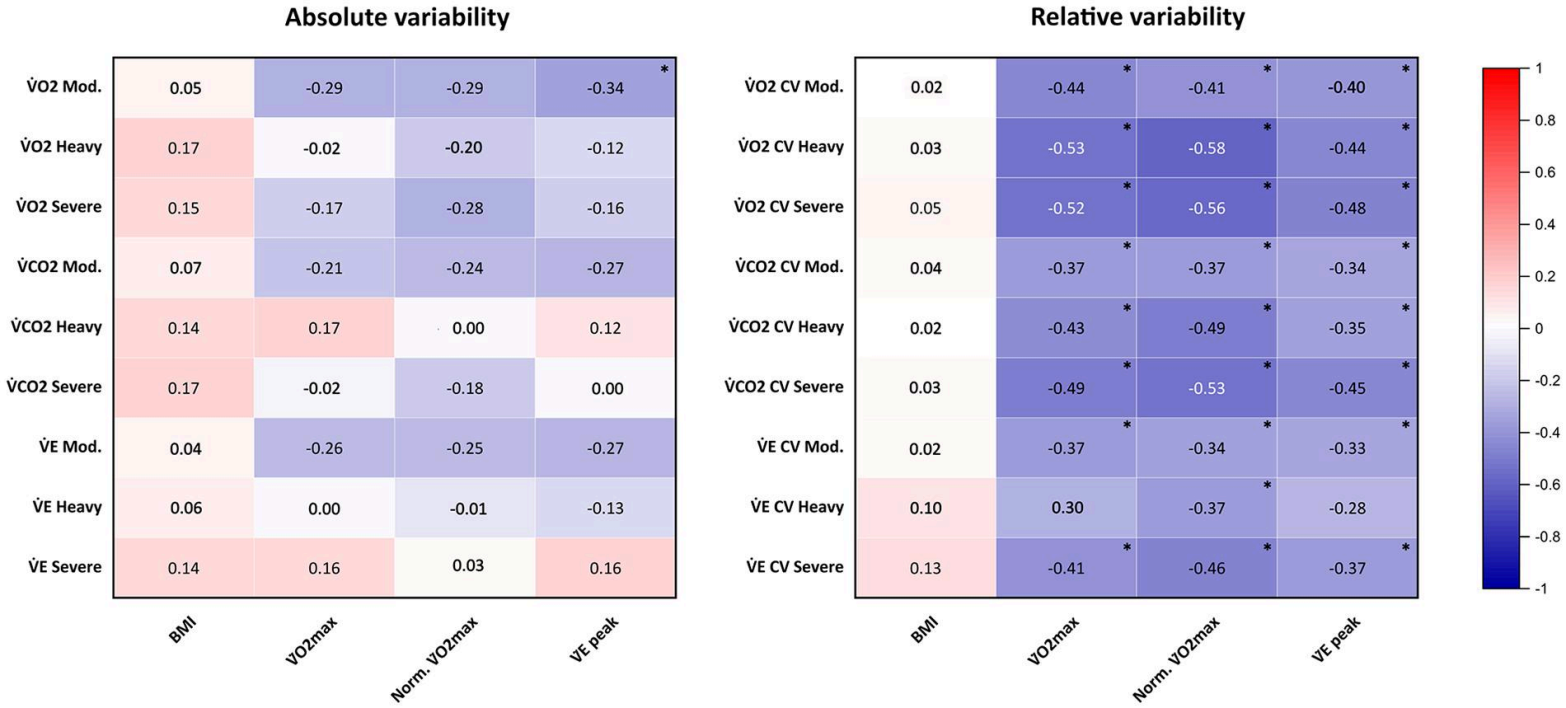
Correlation matrices between participants’ characteristics and absolute (left) and relative variability (right) in V̇O_2_, V̇CO_2_, and V̇E, measured respectively in the moderate, heavy, and severe domains of exercise.

Correlations between participant characteristics and variability indices are presented in Figure 2. Absolute within-trial variability (SD) showed no consistent association with anthropometric or functional measures. In contrast, relative within-trial variability (CV%) demonstrated moderate negative correlations with aerobic fitness indicators (V̇O_2max_, normalised V̇O_2max_, V̇E_peak_), particularly in the heavy and severe domains (*r*∼−0.34 to −0.58, *p* < 0.05).

Linear mixed-effects modelling (full output in Supplementary Table S2) showed that exercise intensity expressed as %RCP was associated with absolute and relative variability. For V̇O_2_, absolute within-trial variability (SD) was not associated with intensity (*β* = 0.063, 95% CI −0.076 to 0.203, *p* = 0.373), whereas relative within-trial variability (CV%) decreased significantly with increasing %RCP (*β* = −0.0004 per %RCP, 95% CI −0.001 to 0.001, *p* < 0.001). For V̇CO_2_, SD increased with intensity (*β* = 0.336 mL·min^−1^ per %RCP, 95% CI 0.193 to 0.479, *p* < 0.001), while CV% decreased (*β*∼−0.001 per %RCP, *p* < 0.001). Similarly, V̇E exhibited a significant increase in SD (*β* = 0.032 L·min^−1^ per %RCP, 95% CI 0.027 to 0.037, *p* < 0.001) and a decrease in CV% (*β*∼−0.0004 per %RCP, *p* < 0.001). Normalised V̇O_2max_ and sex did not influence SD in any variable. For CV%, higher normalised V̇O_2max_ was associated with lower variability in V̇O_2_ and V̇CO_2_, while sex contributed modestly to CV% differences in V̇O_2_ and V̇CO_2_ but not V̇E. Between-participant differences accounted for approximately one-third of total variance in SD and up to ∼40% in CV%, indicating a substantial stable inter-individual component of day-to-day variability.

**Figure 3:**
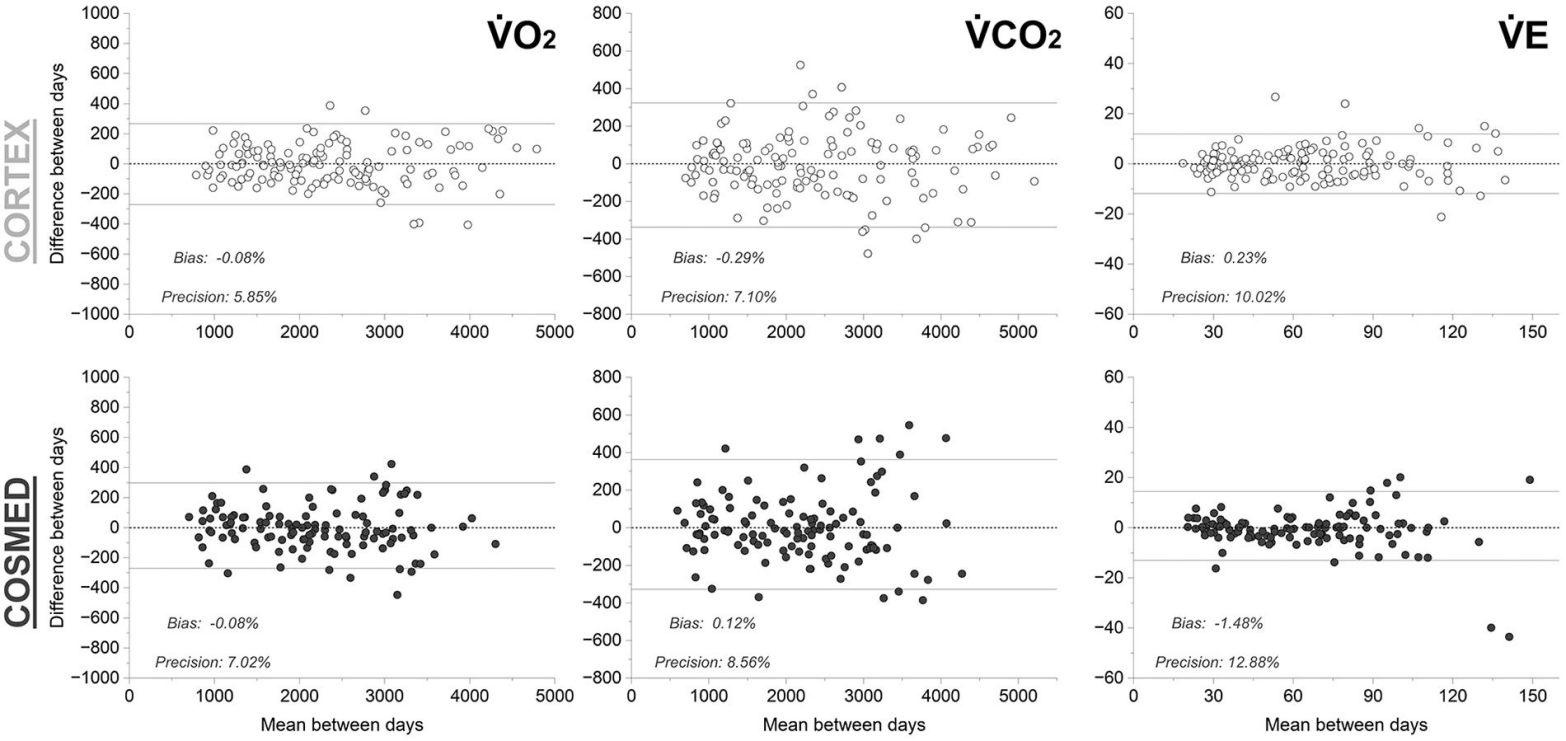
Bland–altman plots showing the agreement between days for V̇O_2_ (left), V̇CO_2_ (middle), and V̇E (right), measured with the CORTEX metaLyzer 3B (top row) and the COSMED quark B2 (bottom row). Each point represents the difference between days as a function of the mean value. Horizontal lines indicate 0 (dotted) and limits of agreement (continued). Reported values indicate the absolute bias and the precision expressed as a percentage.

In contrast to within-trial variability, test–retest reliability assessed from time-averaged values showed that ICCs for V̇O_2_ were excellent (>0.90) at most intensities, with slightly lower values at the lowest work-rates. V̇CO_2_ and V̇E showed reduced test-retest reliability in the moderate domain but improved progressively at higher intensities (Table 3). SEM and MDC followed similar trends, decreasing from moderate to severe domains and confirming improved signal-to-noise characteristics with rising work-rate. For V̇O_2_, MDC ranged approximately from 166 ml·min^−1^ to 416 ml·min^−1^ (Table 3). Bland–Altman plots revealed small biases across all variables, with precision improving as intensity increased. Wider limits of agreement for ventilation reflected greater physiological variability in this measure (Figure 3).

**Table 3:** Between-day reliability of gas exchange and ventilation measurements between days for both metabolic carts at moderate (M1 and M2), heavy (H1 and H2) and severe (S1 and S2) exercise intensities.

		Group CORTEX MetaLyzer 3B			Group COSMED Quark B2		
		ICC (0–1)	SEM (ml·min^−^¹)	MDC (ml·min^−^¹)	ICC (0–1)	SEM (ml·min^−^¹)	MDC (ml·min^−^¹)
V̇O2	M1	0.805 [0.594: 0.914]	75.9	210.4	0.713 [0.410: 0.870]	149.9	415.4
	M2	0.935 [0.853: 0.972]	59.9	165.9	0.920 [0.812: 0.967]	92.0	255.0
	H1	0.984 [0.963: 0.993]	75.5	209.2	0.953 [0.888: 0.981]	107.3	297.3
	H2	0.953 [0.892: 0.980]	137.6	381.4	0.942 [0.861: 0.976]	127.5	353.3
	S1	0.982 [0.957: 0.992]	105.8	293.3	0.960 [0.903: 0.984]	122.4	339.1
	S2	0.986 [0.966: 0.994]	96.2	266.6	0.979 [0.947: 0.992]	87.9	243.6
V̇CO2	M1	0.791 [0.565; 0.910]	71.5	198.2	0.641 [0.270: 0.845 ]	95.7	265.2
	M2	0.850 [0.677; 0.934]	100.6	278.9	0.888 [ 0.744: 0.954]	101.9	282.6
	H1	0.977 [0.947; 0.991]	92.6	256.6	0.936 [0.848: 0.974]	123.3	341.7
	H2	0.942 [0.867; 0.975]	163.2	452.5	0.935 [0.845: 0.973]	138.6	384.2
	S1	0.969 [0.928; 0.987]	149.9	413.1	0.944 [ 0.868: 0.977]	150.1	416.2
	S2	0.989 [0.974; 0.995]	100.0	277.2	0.950 [0.878: 0.980]	139.9	387.7

		ICC (0–1)	SEM (L·min^−^¹)	MDC (L·min^−^¹)	ICC (0–1)	SEM (L·min^−^¹)	MDC (L·min^−^¹)
V̇E	M1	0.631 [0.287; 0.825]	2.7	7.6	0.596 [0.205; 0.824]	3.6	10.1
	M2	0.752 [0.490; 0.889]	3.1	8.6	0.933 [0.842; 0.973]	2.1	6.0
	H1	0.936 [0.854; 0.973]	3.3	9.2	0.918 [0.808; 0.966]	4.1	11.3
	H2	0.847 [0.671; 0.933]	5.6	15.5	0.931 [0.837; 0.972]	3.6	9.9
	S1	0.929 [0.840; 0.970]	5.7	15.9	0.848 [0.661; 0.937]	8.7	24.2
	S2	0.951 [0.887; 0.979]	5.6	15.6	0.888 [0.738; 0.955]	8.3	23.1

Values are presented for intraclass correlation coefficients (ICC, with 95% confidence intervals), standard error of measurement (SEM), and minimal detectable change (MDC).

## Discussion

This study examined within-trial signal variability and test–retest reliability of pulmonary gas-exchange and ventilatory variables across the full exercise-intensity continuum (i.e., moderate, heavy, and severe domains). We assessed the influence of both exercise intensity and participants' characteristics on the absolute (i.e., within participant SD) and relative (i.e., CV%) variability of respiratory signals, using two validated metabolic systems under tightly standardised conditions. Overall, variability improved with increasing intensity, with consistent reductions in CV% and generally stable absolute variability for V̇O_2_. By contrast, V̇CO_2_ and V̇E showed slightly greater absolute variability at higher work-rates, suggesting that ventilatory and metabolic responses expand in amplitude but become proportionally more stable. Exercise intensity thus emerged as the main factor associated with measurement stability, while participant characteristics showed limited independent contribution once between-participant variability was accounted for. These results support the initial hypothesis that higher intensities yield smaller physiological variability and provide new domain-specific benchmarks to guide the interpretation and design of repeated-measures studies in exercise physiology.

The participants included in this study were young healthy individuals with a mean V̇O_2max_ of 49.6 ± 8.8 ml·kg^−1^·min^−1^, values that indicate a moderate to high level of aerobic fitness (37). No relevant differences were observed between the two subgroups assigned to the CORTEX and COSMED metabolic carts in terms of anthropometric or functional characteristics, confirming the comparability of the two samples. GET and RCP occurred respectively at 63 ± 5% and 86 ± 5% of V̇O_2max_, which aligns with typical ranges for non-elite, aerobically trained individuals (38, 39).

As expected, V̇O_2_, V̇CO_2_, and V̇E increased with intensity, and no significant differences were observed between days at any work-rate, confirming stable physiological responses across sessions. A possible effect of day was detected for V̇E in the COSMED group, without a corresponding intensity × day interaction. This observation is consistent with previous evidence indicating greater variability in ventilation compared to V̇O_2_ and V̇CO_2_ in human responses (16). However, this effect was not consistent across variables or systems and is therefore unlikely to reflect a systematic bias. This general stability is notable given recent concerns regarding the consistency of breath-by-breath systems under both simulated and human testing (17). In fact, while breath-by-breath analysis is widely used in both clinical and sports contexts, its variability across clearly defined intensity domains has rarely been documented. Most prior studies examined either a single outcome such as V̇O_2max_ (40), device (41) or a specific intensity/protocol (42), and have reported device-related errors generally ranging from ∼1%–8% depending on the system, protocol, exercise intensity, and analytical approach (17–19, 42–44), with some studies reporting even values up to 12% (44). However, to our knowledge, only one study directly compared the systems used in the present study (17) reporting slightly better, yet comparable, performance between metabolic carts. In this context, simulator-based studies and repeated human exercise studies address partially different questions, as the latter additionally incorporate biological variability. Accordingly, our findings should not be interpreted as a formal cross-validation between systems, but rather as intensity-specific reference values for the combined physiological and technical variability encountered during repeated constant-work-rate exercise in humans. Our results align with previous research emphasising the importance of multiple repetitions when assessing gas exchange, particularly at moderate intensities where within-trial signal variability is higher. To our knowledge, this is the first study to provide a domain-specific assessment of reproducibility in V̇O_2_, V̇CO_2_, and V̇E using multiple validated commercial systems under tightly standardized conditions.

Despite the general lack of statistical differences between testing days, visual inspection of individual responses revealed a degree of variability in gas exchange measurements (Figure 1 and Table 2). Specifically, variability was most evident in V̇O_2_ and V̇E at M1 and M2, where fluctuations across days appeared larger in relative terms (e.g., up to ∼350 ml·min^−1^ difference in V̇O_2_ and ∼15 L·min^−1^ in V̇E for some individuals). Moreover, the mean within-trial CV% decreased consistently with increasing exercise intensity for all measured variables (Table 2). The highest CVs were observed at the lowest moderate intensities, reaching up to 11.0% for V̇O_2_ and 11.7% for V̇E, while values below 5% were typical in the severe domain.

Furthermore, simple correlations revealed consistent negative associations between CV% and indicators of aerobic fitness (V̇O_2max_, normalised V̇O_2max_, and V̇E_peak_, Figure 2), suggesting that fitter individuals tended to exhibit lower relative variability in gas-exchange variables. In the mixed-effects models, normalised V̇O_2max_ remained independently associated with CV% for V̇O_2_ and V̇CO_2_, whereas no influence was observed on absolute variability. This pattern indicates that the reduction in CV% with increasing fitness is primarily consistent with scaling effects related to larger mean responses, although a modest independent contribution of aerobic capacity to relative signal behaviour, potentially mediated by differences in ventilatory control or breathing pattern, cannot be excluded. Importantly, these associations were confined to relative variability and did not extend to absolute fluctuations. Rather, exercise intensity increases slightly the absolute variability for V̇CO_2_ and V̇E, but not V̇O_2_, while relative variability consistently decreases. From a practical standpoint, this asymmetry between V̇O_2_ and V̇CO_2_ variability implies that variables derived from their ratio, such as the respiratory exchange ratio or substrate oxidation estimates, may exhibit greater uncertainty at higher intensities and require additional caution in their interpretation. Our results are also coherent with findings from Hopker et al. (45), who reported that gross efficiency measured via the Douglas bag method yields more stable estimates at higher work-rates (45). Within-trial signal variability appears greatest in the moderate domain, where lower ventilatory and metabolic demands may amplify the influence of spontaneous breathing variation or measurement artifacts. By contrast, heavy and severe domains showed reduced relative variability, suggesting they may provide more stable windows for assessing submaximal physiological function, provided that their more complex kinetics are properly accounted for (21, 22). Nevertheless, substantial interindividual variability remained evident, indicating that some individuals could consistently exhibit more stable ventilatory and metabolic responses than others across repeated exercise sessions.

Regarding the reliability of the respiratory signals, ICC values remained generally excellent across intensities and variables, especially in the heavy and severe domains, where most approached 0.95 (Table 3). At M1, lower ICCs were observed, in line with the reduced signal amplitude and proportionally greater influence of breath-by-breath fluctuations. SEM values followed the same pattern, ranging from ∼60 to ∼140 ml·min^−1^ for V̇O_2_, depending on intensity and device. These values reflect the between-day stability of time-averaged pulmonary gas-exchange measurements under constant work-rate conditions when assessed within individuals across days, and they align with previous estimates of technical accuracy obtained under simulated conditions (17, 19). Taken together, these results support the positive conclusions of Thiessen et al. and reinforce the test-retest reliability of gas exchange variables under well-standardised conditions.

One of the main goals of this study was to provide domain-specific values for statistical design and physiological interpretations. In this context, while a few studies have evaluated MDC in portable metabolic carts using a metabolic simulator (COSMED K5) (14) or during walking (COSMED K4b2) (46), our study extends this knowledge by providing intensity-domain-specific MDC values under steady-state conditions, accounting for both technical and physiological variability. Across intensity domains, the MDC for V̇O_2_ ranged approximately from 166 to 416 ml·min^−1^, depending on work-rate and system (Table 3). These thresholds indicate that physiological changes not exceeding ∼250–400 ml·min^−1^ should be interpreted with caution, especially when other forms of bias are present (e.g., non-blind study designs). From an applied perspective, this suggests that small changes in V̇O_2_ measured during repeated CPET assessments (below 250 to 400 ml·min^−1^, specific intensity-specific values are reported in Table 3), particularly in less fit individuals or at lower exercise intensities, may fall within the expected range of combined physiological and technical variability. In these contexts, interpretation of longitudinal changes may benefit from integrating additional physiological information beyond an isolated V̇O_2_ value, including exercise kinetics, ventilatory responses, heart rate behaviour, and external work-rate responses.

Finally, the consistency of gas exchange measurements was further supported by Bland–Altman analysis, which confirmed the absence of systematic bias between test days with mean differences close to zero for both V̇O_2_ and V̇CO_2_. Slightly narrower limits of agreement and better precision overall, particularly for V̇E, were recorded in the CORTEX group.

## Limitations

This study has a few limitations that should be considered. First, the use of two independent metabolic carts without cross-over testing between systems should be acknowledged. Because participants were tested exclusively on one system, direct inter-device agreement could not be formally evaluated. Although identical calibration procedures, ergometer models, testing protocols, and data processing methods were applied across systems, potential systematic differences between platforms cannot be excluded. Therefore, findings should be interpreted within-device rather than as evidence of cross-system equivalence, and specific to young, healthy adults**.** Second, each constant work-rate trial lasted six minutes, which is generally sufficient to reach steady state in moderate and heavy domains but may not capture longer-term physiological variability or progressive ventilatory drift. Additionally, the analysis focused on phase III of V̇O_2_ kinetics but was not aimed at exploring phases I and II, limiting insight into dynamic responses. Another methodological consideration relates to the use of a single MRT-based correction to translate ramp-derived responses into constant-work-rate exercise intensities. Recent work has highlighted that MRT is unlikely to represent a fixed physiological constant across exercise intensities and conditions (32), which may limit the precision of this type of adjustment approach. Nevertheless, in the present study, this correction was applied systematically within participants using the same procedure across all conditions, thereby reducing random methodological variability. In addition, the selected exercise intensities (33%*Δ* and 66%*Δ* within each domain) were intentionally positioned sufficiently within the moderate, heavy, and severe domains to minimise the likelihood that small errors in MRT correction would alter domain allocation. Despite these precautions, some residual uncertainty in the precise estimation of target work rates cannot be excluded.

Finally, although variability metrics were derived from all interpolated and 5 s averaged data points within the final minute of exercise rather than from a single 1 min average value, the processing approach used in the present study still represents a smoothed representation of the original breath-by-breath signal. This approach was intentionally chosen to improve comparability across exercise intensities by partially reducing the influence of differing breathing frequencies and irregular breath timing. However, it may also attenuate some short-term physiological fluctuations compared with analyses performed directly on raw breath-by-breath data.

## Conclusions

This study provides a comprehensive assessment of within-trial signal variability and day-to-day reliability of pulmonary gas-exchange and ventilation across defined exercise-intensity domains. We described the potential contribution of participants' characteristics and exercise intensity to the absolute and relative variability of respiratory signals. Overall, CV% decreased with increasing intensity, whereas absolute variability remained stable for V̇O_2_ and increased modestly for V̇CO_2_ and V̇E. Exercise intensity emerged as the dominant factor associated with within-trial variability across exercise intensities. However, normalised V̇O_2max_ and, to a lesser extent, sex explained a modest proportion of variance in relative variability (CV%) for V̇O_2_ and V̇CO_2_, while no participant characteristic influenced absolute variability. Thus, although intensity primarily governs measurement stability, fitness-related scaling effects contribute secondarily to relative variability. The derived SEM and MDC values provide practical thresholds to support the interpretation of physiological changes and domain-specific benchmarks for reliability that can inform methodological and applied physiological interpretation in health, performance, and research contexts.

## Data Availability

The raw data supporting the conclusions of this article will be made available by the authors, upon request to the corresponding author without undue reservation.
