# Correction: Exercise intensity influences variability and test-retest reliability of pulmonary gas-exchange measurements during constant work-rate cycling

**DOI:** 10.3389/fspor.2026.1940821

**Published:** 2026-07-30

**Authors:** Alessandro L. Colosio, Massimo Teso, Maura Loi, Lena Stuer, Jan Boone, Silvia Pogliaghi

**Affiliations:** 1 Laboratoire Interuniversitaire de Biologie de la Motricité, Université Jean Monnet Saint-Etienne, Saint-Étienne, France; 2 College of Health and Life Sciences, Hamad Bin Khalifa University, Doha, Qatar; 3 Department of Neurosciences, Biomedicine and Movement Sciences, University of Verona, Verona, Italy; 4 Department of Movement and Sports Sciences, Ghent University, Ghent, Belgium; 5 Department of Clinical and Experimental Medicine, University of Foggia, Foggia, Italy

**Keywords:** carbon dioxide, CORTEX metaLyzer 3B, COSMED quark B2, indirect calorimetry, oxygen consumption, V̇o2

The approximation symbol (∼) was inadvertently rendered as a minus sign (−) in four instances.

A correction has been made to the **Discussion** section, Paragraph 3. The corrected sentence is as follows:

“Most prior studies examined either a single outcome such as V̇O_2max_ (40), device (41) or a specific intensity/protocol (42), and have reported device-related errors generally ranging from ∼1%–8% depending on the system, protocol, exercise intensity, and analytical approach (17–19, 42–44), with some studies reporting even values up to 12% (44).”

A correction has been made to the **Discussion** section, Paragraph 4. The corrected sentence is as follows:

“Specifically, variability was most evident in V̇O_2_ and V̇E at M1 and M2, where fluctuations across days appeared larger in relative terms (e.g., up to ∼350 mL·min^−1^ difference in V̇O_2_ and ∼15 L·min^−1^ in V̇E for some individuals).”

A correction has been made to the **Discussion** section, Paragraph 6. The corrected sentence is as follows:

“SEM values followed the same pattern, ranging from ∼60 to ∼140 mL·min^−1^ for V̇O_2_, depending on intensity and device.”

A correction has been made to the **Discussion** section, Paragraph 7. The corrected sentence is as follows:

“These thresholds indicate that physiological changes not exceeding ∼250–400 mL·min^−1^ should be interpreted with caution, especially when other forms of bias are present (e.g., non-blind study designs).”

Some grammatical issues were identified in the published version.

A correction has been made to the section **Methods**, “Protocol”, Paragraph 1. The corrected sentence is as follows:

“Each CWR started with 1 min of seated rest on the ergometer, followed by 3 min unloaded cycling at 30 revolutions per minute (rpm) performed to standardise the exercise onset.”

A correction has been made to the section **Methods**, “Protocol”, Paragraph 2. The corrected sentence is as follows:

“Participants were instructed to maintain their habitual physical activity patterns throughout the study period and were informally monitored regarding changes in training routine besides the testing sessions.”

A correction has been made to the section **Methods**, “Ramp incremental test”, Paragraph 3. A sentence was duplicated: “These values were used to determine the intensities of successive tests.” The first occurrence has been deleted.

A correction has been made to the **Discussion** section, Paragraph 3. The corrected sentence is as follows:

“In this context, simulator-based studies and repeated human exercise studies address partially different questions, as the latter additionally incorporate biological variability.”

The original version of this article has been updated.

