# Supplementary material for: Exercise intensity influences variability and test-retest reliability of pulmonary gas-exchange measurements during constant work-rate cycling
Source: Front Sports Act Living. 2026 Jun 22;8:1814527. doi: 10.3389/fspor.2026.1814527 (PMC13333782; doi:10.3389/fspor.2026.1814527)
Supplement: Supplementary file 1 [file Table1.docx]

|  | **System** | **Intensity** | **Day** | **Intensity × Day** |
| --- | --- | --- | --- | --- |
| **VO2** | CORTEX | F(5,105) = 113.70, p < 0.001 | F(1,21) = 0.02, p = 0.89 | F(5,105) = 1.17, p = 0.33 |
|  | COSMED | F(5,90) = 192.97, p < 0.001 | F(1,18) = 1.32, p = 0.27 | F(5,90) = 0.58, p = 0.72 |
| **VCO2** | CORTEX | F(5,105) = 127.26, p < 0.001 | F(1,21) = 0.41, p = 0.53 | F(5,105) = 0.77, p = 0.52 |
|  | COSMED | F(5,90) = 230.14, p < 0.001 | F(1,18) = 0.13, p = 0.72 | F(5,90) = 0.41, p = 0.84 |
| **VE** | CORTEX | F(5,105) = 138.26, p < 0.001 | F(1,21) = 0.02, p = 0.89 | F(5,105) = 0.65, p = 0.66 |
|  | COSMED | F(5,90) = 136.70, p < 0.001 | F(1,18) = 4.92, p = 0.04 | F(5,90) = 0.52, p = 0.76 |

**Table 1S** Two-way repeated-measures ANOVA (day x intensity) results for V̇O2, VCO2 and VE average values measured during the last minute of the 6 min constant work rate trials.
