# Supplementary material for: Exercise intensity influences variability and test-retest reliability of pulmonary gas-exchange measurements during constant work-rate cycling
Source: Front Sports Act Living. 2026 Jun 22;8:1814527. doi: 10.3389/fspor.2026.1814527 (PMC13333782; doi:10.3389/fspor.2026.1814527)
Supplement: Supplementary file 2 [file Table2.docx]

|  | **SD (absolute variability)** | | | | | | | | | | | **CV% (relative variability)** | | | | | | | | | | | | | |
| --- | --- | --- | --- | --- | --- | --- | --- | --- | --- | --- | --- | --- | --- | --- | --- | --- | --- | --- | --- | --- | --- | --- | --- | --- | --- |
|  | **Predictor** | **β** | **SE** | **95% CI** | **t** | **p** | **Variance** | | | **β** | | | **SE** | | | **95% CI** | | | **t** | **p** | **Variance** | | | |  |
| **V̇O₂** | %RCP | 0.063 | 0.071 | -0.076 to 0.203 | 0.891 | 0.373 | Random intercept variance | 950 | | -0.0004 | | | 0.000 | | | -0.001 to 0.001 | | | -14.005 | **p < 0.001** | Random intercept variance | 0.0003 | | |  |
|  | normVO₂max | -0.937 | 0.665 | -2.244 to 0.370 | -1.409 | 0.159 | Residual variance | 1633 | | -0.001 | | | 0.000 | | | -0.002 to 0.000 | | | -2.493 | **0.013** | Residual variance | 0.0006 | | |  |
|  | sex | -12.463 | 11.582 | -35.219 to 10.293 | -1.076 | 0.282 | ICC | 37% | | -0.013 | | | 0.007 | | | -0.029 to -0.003 | | | -2.434 | **0.015** | ICC | 32% | | |  |
|  | **SD (absolute variability)** | | | | | | | | | | | **CV% (relative variability)** | | | | | | | | | | | | | |
|  | **Predictor** | **β** | **SE** | **95% CI** | **t** | **p** | **Variance** | | | | | **β** | | | **SE** | | | **95% CI** | **t** | **p** | **Variance** | |  |  |  |
| **V̇CO₂** | %RCP | 0.336 | 0.073 | 0.193 to 0.479 | 4.602 | **p < 0.001** | Random intercept variance | | 798 | | -0.001 | | | 0.000 | | | -0.001 to -0.001 | | -15.394 | **p < 0.001** | Random intercept variance | 0.0003 | |  |  |
|  | normVO₂max | -0.677 | 0.618 | -1.892 to 0.538 | -1.094 | 0.274 | Residual variance | | 1718 | | -0.001 | | | 0.000 | | | -0.002 to 0.000 | | -2.132 | **0.033** | Residual variance | 0.0005 | |  |  |
|  | sex | -3.246 | 10.77 | -24.406 to 17.913 | -0.301 | 0.763 | ICC | | 32% | | -0.014 | | | 0.007 | | | -0.027 to -0.001 | | -2.146 | **0.032** | ICC | 41% | |  |  |
|  | **SD (absolute variability)** | | | | | | | | | | | **CV% (relative variability)** | | | | | | | | | | | | | |
|  | **Predictor** | **β** | **SE** | **95% CI** | **t** | **p** | **Variance** | | | | | **β** | | | **SE** | | | **95% CI** | **t** | **p** | **Variance** | |  |  |  |
| **V̇E** | %RCP | 0.032 | 0.003 | 0.027 to 0.037 | 12.773 | **p < 0.001** | Random intercept variance | | 1.196 | | -0.0004 | | | 0.000 | | | -0.001 to -0.002 | | -9.033 | **p < 0.001** | Random intercept variance | 0.0005 | |  |  |
|  | normVO₂max | -0.016 | 0.024 | -0.062 to 0.031 | -0.659 | 0.51 | Residual variance | | 2.028 | | -0.001 | | | 0.000 | | | -0.002 to 0.000 | | -1.8 | 0.072 | Residual variance | 0.0008 | |  |  |
|  | sex | 0.025 | 0.411 | -0.781 to 0.832 | 0.062 | 0.951 | ICC | | 37% | | -0.014 | | | 0.009 | | | -0.031 to 0.003 | | -1.581 | 0.114 | ICC | 40% | |  |  |

**Table 2S. Linear mixed-effects models examining determinants of absolute (SD) and relative (CV%) variability in V̇O₂, V̇CO₂ and V̇E**
